# Comparable roles for serotonin in rats and humans for computations underlying flexible decision-making

**DOI:** 10.1038/s41386-023-01762-6

**Published:** 2023-11-01

**Authors:** Qiang Luo, Jonathan W. Kanen, Andrea Bari, Nikolina Skandali, Christelle Langley, Gitte Moos Knudsen, Johan Alsiö, Benjamin U. Phillips, Barbara J. Sahakian, Rudolf N. Cardinal, Trevor W. Robbins

**Affiliations:** 1 National Clinical Research Center for Aging and Medicine at Huashan Hospital, State Key Laboratory of Medical Neurobiology and Ministry of Education Frontiers Center for Brain Science, Institutes of Brain Science and Institute of Science and Technology for Brain-Inspired Intelligence, Fudan University, Shanghai, 200433 P. R. China; 2 Center for Computational Psychiatry, Ministry of Education Key Laboratory of Computational Neuroscience and Brain-Inspired Intelligence, Human Phenome Institute, Fudan University, Shanghai, 200433 China; 3 Department of Psychology, University of Cambridge, Cambridge, CB2 3EB UK; 4 Behavioural and Clinical Neuroscience Institute, University of Cambridge, Cambridge, CB2 3EB UK; 5 Aelis Farma, 33077 Bordeaux, France; 6 Department of Psychiatry, University of Cambridge, Cambridge, CB2 0SZ UK; 7 Cambridgeshire and Peterborough NHS Foundation Trust, Cambridge, CB21 5EF UK; 8 NIHR Cambridge Biomedical Research Centre, University of Cambridge, Cambridge, CB2 0QQ UK; 9 Neurobiology Research Unit, the Neuroscience Centre, Copenhagen University Hospital Rigshospitalet, Copenhagen, Denmark; 10 Department of Clinical Medicine, University of Copenhagen, Copenhagen, Denmark

**Keywords:** Cognitive control, Transporters in the nervous system

## Abstract

Serotonin is critical for adapting behavior flexibly to meet changing environmental demands. Cognitive flexibility is important for successful attainment of goals, as well as for social interactions, and is frequently impaired in neuropsychiatric disorders, including obsessive–compulsive disorder. However, a unifying mechanistic framework accounting for the role of serotonin in behavioral flexibility has remained elusive. Here, we demonstrate common effects of manipulating serotonin function across two species (rats and humans) on latent processes supporting choice behavior during probabilistic reversal learning, using computational modelling. The findings support a role of serotonin in behavioral flexibility and plasticity, indicated, respectively, by increases or decreases in choice repetition (‘stickiness’) or reinforcement learning rates following manipulations intended to increase or decrease serotonin function. More specifically, the rate at which expected value increased following reward and decreased following punishment (reward and punishment ‘learning rates’) was greatest after sub-chronic administration of the selective serotonin reuptake inhibitor (SSRI) citalopram (5 mg/kg for 7 days followed by 10 mg/kg twice a day for 5 days) in rats. Conversely, humans given a single dose of an SSRI (20 mg escitalopram), which can decrease post-synaptic serotonin signalling, and rats that received the neurotoxin 5,7-dihydroxytryptamine (5,7-DHT), which destroys forebrain serotonergic neurons, exhibited decreased reward learning rates. A basic perseverative tendency (‘stickiness’), or choice repetition irrespective of the outcome produced, was likewise increased in rats after the 12-day SSRI regimen and decreased after single dose SSRI in humans and 5,7-DHT in rats. These common effects of serotonergic manipulations on rats and humans—identified via computational modelling—suggest an evolutionarily conserved role for serotonin in plasticity and behavioral flexibility and have clinical relevance transdiagnostically for neuropsychiatric disorders.

## Introduction

Humans and other animals alike must maximise rewards and minimise punishments to survive and thrive. Across phylogeny this involves learning about cues or locations that inform whether an action is likely to result in a good or bad outcome. Adaptive behavior, however, must also be flexible: the ability to disengage from previously learned actions that are no longer useful or appropriate to the situation is fundamental to well-being. Indeed, behavior can become abnormally stimulus-bound and perseverative in compulsive disorders [1–5]. Furthermore, learning the best course of action can require withstanding occasional negative feedback, which should sometimes be ignored if rare. Indeed, inappropriately switching behavior away from an adaptive action following misleading or even negative feedback (‘lose-shift’) has been reported across several traditional psychiatric diagnostic categories [6–10].

**Fig. 1:**
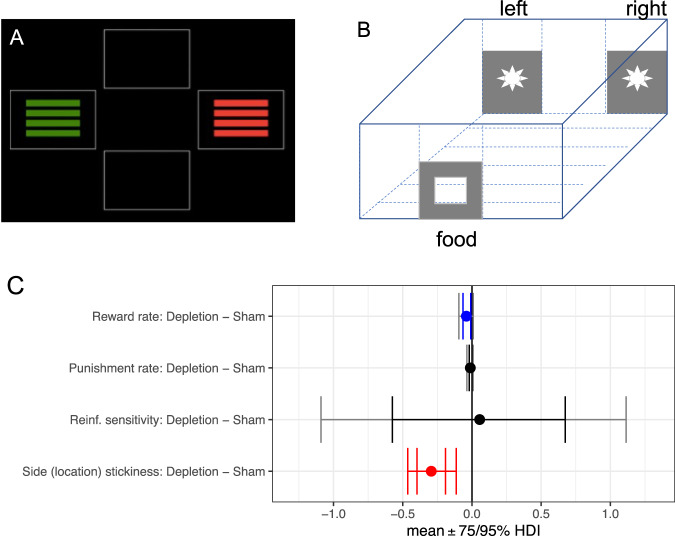
Task schematics for probabilistic reversal learning and effects of serotonin depletion on model parameters in rats. **A** Experiment in humans (example trial on touchscreen computer) and (**B**) Experiment in rats (two apertures illuminated simultaneously to the left and right of a central aperture with reinforcement contingencies 80% : 20% for left : right or right : left, and a food pellet was given to a food magazine positioned on the opposite wall of the operant chamber if reward was delivered). **C** Side (location) stickiness was diminished by neurotoxic 5-HT depletion, i.e., 5,7-dihydroxytryptamine. Reinf. reinforcement. Red signifies a difference between the parameter per-condition mean according to the Bayesian “credible interval”, 0 ∉ 95% HDI. Blue signifies 0 ∉ 75% HDI. The inner interval represents the 75% HDI, while the outer interval represents the 95% HDI.

The neurotransmitter serotonin (5-hydroxytryptamine; 5-HT) is widely implicated in behavioral flexibility [11–19]. Perturbing 5-HT function can affect both perseveration and lose-shift behavior, which are commonly assessed using probabilistic reversal learning (PRL) paradigms (Fig. 1A, B): a subject learns through trial and error the most adaptive action in a choice procedure, the contingencies of which eventually reverse, sometimes repeatedly [12, 20–22]. A unifying framework for 5-HT in these processes has, however, remained elusive. To this end, we proposed to use a mechanistic modelling framework to align behavioral changes in PRL following serotonergic manipulations in rats [20] and humans [23].

Two common methods for studying serotonin are through serotonin depletion and treatment with selective serotonin reuptake inhibitors (SSRIs). In non-human animals, depletion can be achieved using the neurotoxin 5,7-dihydroxytryptamine (5,7-DHT) which produces a profound loss of serotonergic fibers [24]. SSRIs, meanwhile, are first-line pharmacological treatments for several psychiatric conditions including major depressive disorder (MDD) [25] and obsessive–compulsive disorder (OCD) [26], yet both the neural and computational mechanisms underlying their efficacy remain poorly understood. Although SSRIs increase extracellular serotonin levels via blocking the reuptake of 5-HT, acute administration of SSRIs, especially at low doses, can reduce 5-HT neurotransmission and chronic administration is often necessary to produce increases in serotonin levels [27, 28]. For this reason, effects of both acute and chronic SSRIs in rats were studied, with the hypothesis that a higher acute dose or chronic administration could overcome these feedback effects of a low acute dose and produce an increase in serotonin transmission [20]. An acute low (e.g. 1 mg/kg) dose of an SSRI such as citalopram increases levels of 5-HT in the dorsal raphe [29] and inhibits the firing of 5-HT neurons due to inhibitory autoreceptor activation there [30, 31] whereas a higher dose (e.g. 10 mg/kg) increases 5-HT levels in the rat prefrontal cortex [29]. Indeed, opposite effects on 5-HT transmission and hence behavior of low and high doses of SSRIs have been reported [20, 32]. Acute administration of 20 mg escitalopram has been shown to reduce cortical 5-HT levels in humans [23] and so we also investigated effects of chronic escitalopram [33].

Reinforcement learning (RL) is a well-established computational mechanism underlying adaptive behavior. It often incorporates the value-based parameters that estimate how quickly action values are learned after receiving reward (‘reward learning rate’, *α*^*rew*^) or punishment (‘punishment learning rate’, *α*^*pun*^) and the extent to which that value is acted upon (often termed ‘inverse temperature’ in relation to the mathematical softmax function typically used; here, termed ‘reinforcement sensitivity’, *τ*^*reinf*^) [34]. This is consistent with the traditional view of stimulus–response habits–that they are created and strengthened by reinforcement [35]. Recently, other aspects of behavior that are independent of reinforcement or value, especially value-free (action outcome-irrelevant) factors were shown to be important for understanding goal-directed decision-making [36], but have been notably absent from prominent computational accounts of goal-directed (or ‘model-based’) versus habitual (or ‘model-free’) controllers of behavior [36–38].

To model the value-free component of behavior, stickiness parameters have been introduced to the RL models to track the extent to which behavioral tendencies are shaped by engagement with discrete cues (stimuli) or locations, irrespective of an action’s outcome [37, 39]. Indeed, stickiness has been found to be significantly higher in stimulant use disorder (SUD) but abnormally low in obsessive–compulsive disorder (OCD) during PRL performance [7]. Therefore, accounting for stickiness—value-free perseveration—may therefore aid in better dissecting the nature of imbalanced goal-directed versus habitual behavior seen in OCD, SUD, and other conditions [40–42], a balance that is sensitive to serotonergic disruption in humans and rodents [43–45]. Across six previously published behavioral experiments in rats [20] and humans [23] and a recently published computational modelling study in humans [33], we examined whether stickiness or other RL parameters (learning rates or reinforcement sensitivity) contributed meaningfully to behavior, and examined whether 5-HT function would consistently modulate any of these parameters across species.

The primary question was whether serotonergic manipulations would cause similar cross-species perturbations of model parameters, thereby demonstrating the evolutionary significance of the role of serotonin in cognitive flexibility. As an increased tendency for lose-shift behavior induced by an acute SSRI has been conceptualised as hypersensitivity to negative feedback [20, 23], we asked whether this would be reflected in elevated punishment learning rates. Selective 5-HT depletion via 5,7-DHT of the orbitofrontal cortex (OFC) or amygdala in marmoset monkeys, meanwhile, reduced reinforcement learning rates (for rewards or punishments), and modulated stickiness [46]; we hypothesised that changes in learning rate or stickiness parameters would occur following global 5-HT manipulations in rats and humans. We predicted that incorporating stickiness parameters would be central to capturing effects of 5-HT on behavioral flexibility and would increase or decrease depending on changes in serotonin transmission.

## Methods

### Probabilistic reversal learning task

The task used in the human SSRI experiment [23] contained 80 trials (Fig. 1A). For the first 40 trials, one stimulus yielded positive feedback on 80% of trials, the other stimulus on 20% of trials. These contingencies reversed for the latter 40 trials. Positive or negative feedback was given by a word “CORRECT” or “WRONG” on the touchscreen computer and a high or low tone. The task was self-paced.

Rats were presented with two apertures illuminated simultaneously to the left and right of a central (inactive) aperture (Fig. 1B; [20]). Responding at the ‘correct’ location was associated with food reward on 80% of trials and a time-out punishment on 20% of trials, while responding at the ‘incorrect’ location had a reversed contingency. Reversals occurred after the animal made eight consecutive correct responses, at which point the correct aperture became the incorrect aperture and vice versa. A session consisted of 200 trials to be completed during a 40-minute period. One session was conducted per day.

### 5-HT manipulations in rats

All the animal experiments were conducted in accordance with the United Kingdom Animals (Scientific Procedures) Act, 1986 (PPL 80/2234) in our previous study [20]. In rats, the effects of 5-HT on the probabilistic reversal leaning were systematically manipulated in 3 experiments: (1) 5,7-DHT forebrain 5-HT depletion (*n* = 16), (2) acute SSRI administration of 1 mg/kg or 10 mg/kg (*n* = 11); and (3) repeated (5 mg/kg for 7 consecutive days) and sub-chronic SSRI administration (10 mg/kg twice a day for 5 consecutive days after the 7-day repeated administration) (*n* = 14). In all experiments, the animals were randomly assigned to the group receiving the citalopram manipulation and the control group receiving the vehicle. Details are provided in Supplementary Methods 2 and have been reported previously [20].

### SSRI administration in humans

The protocol was ethically approved (Cambridge Central NHS Research Ethics Committee, reference 15/EE/0004). Volunteers gave informed consent and were paid. Participants were healthy and without a personal or family history of psychiatric or neurological disorders [23]. In a randomised, double-blind, placebo-controlled, between-groups design [23], healthy volunteers received either escitalopram (*n* = 32) or placebo (*n* = 33). The PRL task was conducted following a 3-hour waiting period after oral drug administration to attain peak plasma escitalopram concentration [47]. Details are provided in the Supplementary Methods 2 and have also been reported previously [23].

In another PRL study [33], participants were semi-randomized into a group (*n* = 32) receiving 20 mg chronic escitalopram or the control group (*n* = 34) receiving placebo for 3 to 5 weeks.

### Computational modelling of behavior

Following our previous publication [7], four RL models were fitted to the behavioral data, which incorporated parameters that have been studied previously using a hierarchical Bayesian method [7, 48]. Briefly, model 1 had three parameters, including the *reward learning rate* (*α*^*rew*^), *punishment learning rate* (*α*^*pun*^), and *reinforcement sensitivity* (*τ*^*reinf*^). Model 2 was as model 1 but incorporated a “*stimulus stickiness*” parameter (*τ*^*stim*^), which measures the tendency to repeat a response to a specific perceptual stimulus, irrespective of the action’s outcome. Model 3 was similar to model 2 but with a single learning rate (*α*^*reinf*^). Model 4 was derived from the experienced-weighted attraction model (EWA) [49]. Model specifications are provided in Supplementary Methods 4.

Models were fitted via Hamiltonian Markov chain Monte Carlo sampling implemented in Stan 2.17.2 [50]. Convergence was checked according to $\hat{R}$, the potential scale reduction factor measure [51, 52], which approaches 1 for perfect convergence. Values below 1.2 are typically used as a guideline for determining model convergence and 1.1 is a stringent criterion [51]. Importantly, we used this stringent criterion as a safeguard against arbitrary and incorrect assignment of variance to different parameters [51]. In the current study, most of the models had an $\hat{R} < 1.1$, except for Model 4 in the sub-chronic 10 mg/kg experiment in rats ($\hat{R}=1.7$) and Model 1 in the 5,7-DHT experiment in rats ($\hat{R}=1.5$). We assumed the four models examined had the same prior probability (0.25). Models were compared via a bridge sampling estimate of the likelihood [53], using the “bridgesampling” package in R [54]. Bridge sampling directly estimates the marginal likelihood, and therefore the posterior probability of each model given the data (and prior model probabilities), under the assumption that the models represent the entire group of those to be considered. Posterior distributions were interpreted using the highest density interval (HDI) of posterior distributions, which is the Bayesian “credible interval”, at different posterior probability levels including 75%, 80%, 85%, 90% and 95%. Together with the HDI, the group mean difference (MD) was also reported. Supplementary Table 1 lists the priors used for each parameter. For the human experiments, trials were sequenced across all 80 trials of the PRL task, and on each trial the computational model was supplied with the participant’s identification number and condition, whether the trial resulted in positive or negative feedback, and which visual stimulus was selected. For the rat experiments, trials were sequenced across all sessions conducted under a given manipulation, and the computational model was supplied with the same information, but instead with the location of the aperture selected rather than the identification of the stimulus selected. Omissions were rare and they were not included in the computational analysis. The source code is available at https://github.com/qluo2018/PRLmodeling.

## Results

### Choice of model

Behavior was best described by reinforcement learning models incorporating parameters for stickiness, reinforcement sensitivity, and separate learning rates (Supplementary Table 2), consistent with previous work [7, 48]. The accuracy of parameter recovery was confirmed for this modelling approach previously [7] and by simulations for those parameter values estimated here in each experiment (Supplementary Tables 3–4).

### Serotonin depletion by intraventricular 5,7-dihydroxytryptamine (5,7-DHT): rats

The conventional analysis in the previous publication [20] found a decreased win-stay rate, an increased lose-shift rate and a reduced number of reversals completed in the group of depletion-operated rats (*n* = 8) compared with the group of sham-operated rats (*n* = 8). After computational modelling, we found that the depletion decreased the side (location) stickiness parameter (*τ*^*loc*^; ${{{{{\rm{MD}}}}}}=-0.2938 \, [95 \% {{{{{\rm{HDI}}}}}},-0.4635 \, {{{{{\rm{to}}}}}}-0.1134]$) and the reward learning rate (*α*^*rew*^; ${{{{{\rm{MD}}}}}}=-0.0401 \ [85 \% {{{{{\rm{HDI}}}}}},-0.0757 \ {{{{{\rm{to}}}}}}-0.0033]$; Fig. 1C and Table 1). There was no effect of 5,7-DHT on the punishment learning rate (*α*^*pun*^) or reinforcement sensitivity (*τ*^*reinf*^) ($0\in 75 \% {{{{{\rm{HDI}}}}}}$]. The trends for both an increase of lose-shift rate and the decreases in both win-stay rate and number of reversals in the empirical observations became significant in the simulation of the winning model (Supplementary Result 1 and Supplementary Figs. 1–3), as the model simulations were less noisy and had larger sample sizes (40 vs. 8 per group). Furthermore, because reinforcement sensitivity was also unaffected in Model 1, which did not contain the stickiness parameter, the effect of 5,7-DHT on stickiness was unlikely to be a misattribution of reinforcement sensitivity.

**Table 1:** Summary of learning parameter effects.

	Stickiness *τ*^*stim*^ (humans) *τ*^*loc*^ (rats)	Reward learning rate *α*^*rew*^	Punishment learning rate *α*^*pun*^	Reinf. Sensitivity *τ*^*reinf*^
Rats: neurotoxic depletion of 5-HT	↓***	↓*	−	−
Rats: 1 mg/kg citalopram	↓**	↑**	−	−
Humans: 20 mg escitalopram	↓*	↓**	−	↓..
Rats: 10 mg/kg citalopram	−	↓*	−	↑*
Rats: 5 mg/kg citalopram repeated	↑.	−	↑***	−
Rats: 10 mg/kg citalopram sub-chronic	↑.	↑***	↑***	↓***
Humans: 20 mg escitalopram chronic [33]	−	−	−	↓**

*rew* reward, *pun* punishment, *reinf* reinforcement, *stim* stimulus, *loc* location.

*** stands for 0 ∉ 95% HDI; ** for 0 ∉ 90% HDI; * for 0 ∉ 85% HDI, .. for 0 ∉ 80% HDI, . for 0 ∉ 75% HDI, − for 0 ∈ 75% HDI.

### Acute SSRI: rats

**Fig. 2:**
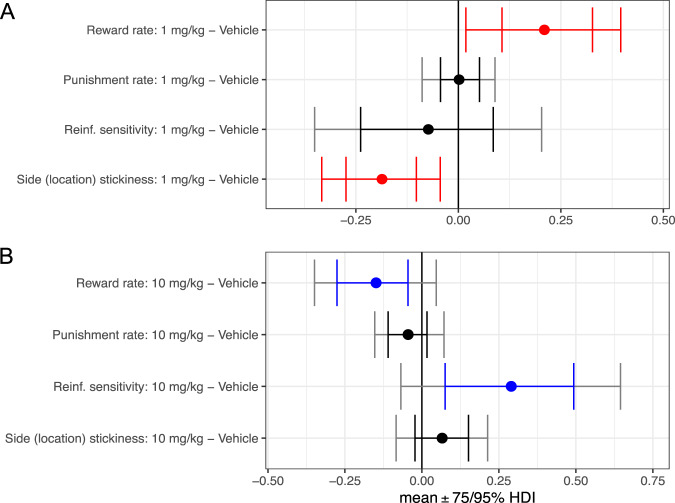
Effects of acute SSRI (citalopram) at two doses on model parameters in rats. **A** for 1 mg/kg and (**B**) for 10 mg/kg. Reinf. reinforcement. mg/kg milligrams per kilogram. Red signifies a difference between the parameter per-condition mean according to the Bayesian “credible interval”, 0 ∉ 95% HDI. Blue signifies 0 ∉ 75% HDI. The inner interval represents the 75% HDI, while the outer interval represents the 95% HDI.

Conventional analysis showed the number of reversals completed was significantly lower following a low dose of 1 mg/kg SSRI compared with a high dose of 10 mg/kg SSRI (*n* = 11 with a cross-over design for vehicle, 1 mg/kg, and 10 mg/kg; [20]). After computational modelling, we found a single dose of 1 mg/kg citalopram in rats diminished the side (location) stickiness parameter $({{{{{\rm{MD}}}}}}=-0.1862 \ [95 \% {{{{{\rm{HDI}}}}}}, -0.3330 \ {{{{{\rm{to}}}}}}-0.0441])$, as seen following 5,7-DHT. The reward learning rate was enhanced by 1 mg/kg in rats (${{{{{\rm{MD}}}}}}=0.2098 \ [95 \% {{{{{\rm{HDI}}}}}},0.0184\,{{{{{\rm{to}}}}}}\,0.3959]$; Fig. 2 and Table 1). There was no effect of 1 mg/kg on punishment learning rate or reinforcement sensitivity ($0\in 75 \% {{{{{\rm{HDI}}}}}}$). A single high dose of citalopram in rats (10 mg/kg) decreased the reward learning rate $({{{{{\rm{MD}}}}}}=-0.1489 \, [85 \% {{{{{\rm{HDI}}}}}},-0.2888 \, {{{{{\rm{to}}}}}}-0.0009])$ and enhanced reinforcement sensitivity (${{{{{\rm{MD}}}}}}=0.2900 \, [85 \% {{{{{\rm{HDI}}}}}},0.0346\,{{{{{\rm{to}}}}}}\,0.5590]$). However, there was no effect of 10 mg/kg on punishment learning rate or side (location) stickiness ($0\in 75 \% {{{{{\rm{HDI}}}}}}$). Simulation of the winning model retrodicted the significantly higher number of  reversals completed in the high-dose group as compared to the low-dose group (Supplementary Result 1 and Supplementary Fig. 4).

### Repeated and sub-chronic SSRI: rats

**Fig. 3:**
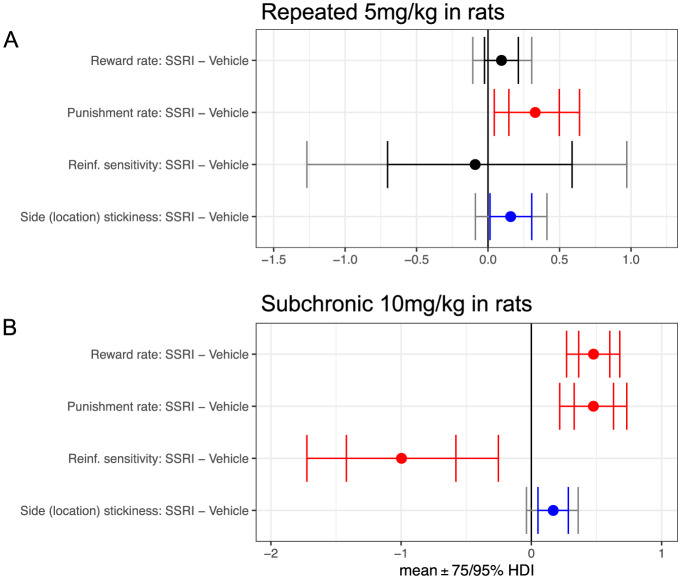
Effects of repeated and sub-chronic SSRI on model parameters in rats. **A** for the repeated SSRI (5 mg/kg citalopram) experiment, and (**B**) for the sub-chronic SSRI (10 mg/kg citalopram) experiment. Reinf. reinforcement. Red signifies a difference between the parameter per-condition mean according to the Bayesian “credible interval”, 0 ∉ 95% HDI. Blue signifies 0 ∉ 75% HDI. The inner interval represents the 75% HDI, while the outer interval represents the 95% HDI.

Conventional analyses showed that win-stay rate increased by repeated 5 mg/kg citalopram administered for consecutive 7 days (the Cit group; *n* = 7) compared with vehicle (Veh; *n* = 7) and the number of reversals was increased by sub-chronic dosing of 10 mg/kg citalopram twice a day for 5 consecutive days in the Cit group after the repeated treatment [20]. Following computational modelling, repeated citalopram enhanced both the punishment learning rate (${{{{{\rm{MD}}}}}}=0.3299 \ [95 \% {{{{{\rm{HDI}}}}}},0.0432\,{{{{{\rm{to}}}}}}\,0.6404]$) and side (location) stickiness (${{{{{\rm{MD}}}}}}=0.1581 \ [75 \% {{{{{\rm{HDI}}}}}},0.0135\,{{{{{\rm{to}}}}}}\,0.3054]$; Fig. 3A and Table 1). There was no effect of repeated citalopram on reward learning rate and reinforcement sensitivity ($0\in 75 \% {{{{{\rm{HDI}}}}}}$). The sub-chronic dosing enhanced the reward learning rate (${{{{{\rm{MD}}}}}}=0.4769 \ [95 \% {{{{{\rm{HDI}}}}}},0.2699\,{{{{{\rm{to}}}}}}\,0.6780]$), punishment learning rate (${{{{{\rm{MD}}}}}}=0.4762 \ [95 \% {{{{{\rm{HDI}}}}}},0.2172\,{{{{{\rm{to}}}}}}\,0.7323]$), and side (location) stickiness (${{{{{\rm{MD}}}}}}=0.1676 \ [75 \% {{{{{\rm{HDI}}}}}},0.0075\,{{{{{\rm{to}}}}}}\,0.3414]$), but decreased reinforcement sensitivity (${{{{{\rm{MD}}}}}}=-0.9972 \ [95 \% {{{{{\rm{HDI}}}}}},-1.7233\, {{{{{\rm{to}}}}}}-0.2540]$; Fig. 3B and Table 1). Again, a trend level increase in the win-stay rate for repeated citalopram in the empirical data became significant in the simulation of the winning model (Supplementary Result 1 and Supplementary Figs. 5–6).

### Acute SSRI: humans

**Fig. 4:**
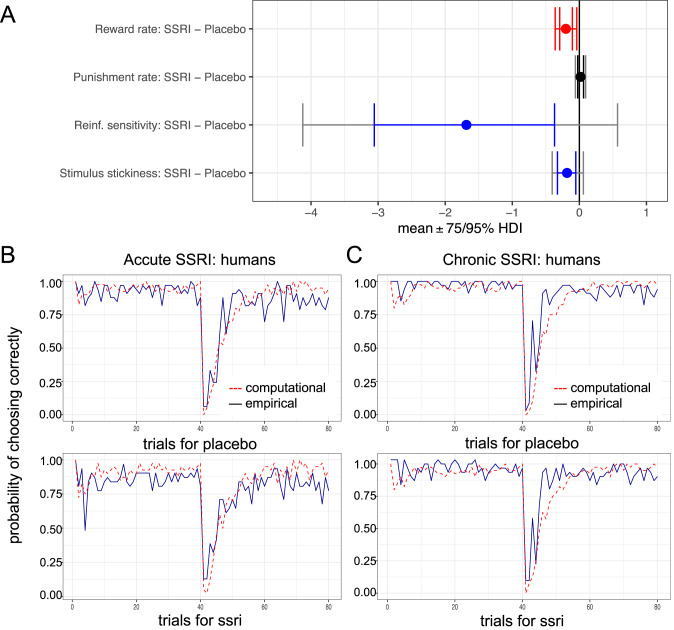
Effects of acute SSRI (20 mg escitalopram) in humans. **A** Stimulus stickiness was decreased following acute SSRI. Reinf.=reinforcement. Red signifies a difference between the parameter per-condition mean according to the Bayesian “credible interval”, 0 ∉ 95% HDI. Blue signifies 0 ∉ 75% HDI. The inner interval represents the 75% HDI, while the outer interval represents the 95% HDI. Trial-by-trial average probability of choosing the optimal stimulus, averaged over subjects during the task in humans following a single 20 mg dose (**B**) and a chronic treatment (**C**) of escitalopram or placebo, or corresponding simulations.

After acute administration of a single 20 mg dose of escitalopram in healthy humans (*n* = 32 escitalopram, *n* = 33 placebo), prior conventional analysis suggested that impaired reversal learning mainly resulted from an elevated lose-shift rate in healthy humans [23]. Computational modelling showed that acute SSRI decreased the reward learning rate $({{{{{\rm{MD}}}}}}=-0.2019 \ [95 \% {{{{{\rm{HDI}}}}}},-0.3612\, {{{{{\rm{to}}}}}}-0.0392])$, stimulus stickiness $({{{{{\rm{MD}}}}}}=-0.1841\left[85 \% {{{{{\rm{HDI}}}}}},-0.3476\,{{{{{\rm{to}}}}}}-0.0045\right])$ and reinforcement sensitivity $({{{{{\rm{MD}}}}}}=-1.6848 \ [80 \% {{{{{\rm{HDI}}}}}}, -3.1501 \, {{{{{\rm{to}}}}}}-0.1553])$, but had no effect on punishment learning rate $(0\in 75 \% {{{{{\rm{HDI}}}}}})$; Fig. 4A and Table 1. Simulation of the computational model retrodicted a significantly increased lose-shift rate (Supplementary Result 1 and Supplementary Fig. 7) and the trial-by-trial average probability of choosing the optimal stimulus (Fig. 4B).

### Chronic SSRI treatment: humans

As reported in our recent publication on effects of chronic SSRI on behavioral flexibility [33], conventional analysis identified no significant group differences in neither the number of losses nor the switch probability [33]. Computational modelling also revealed that chronic SSRI reduced reinforcement sensitivity compared to placebo in healthy volunteers $({{{{{\rm{MD}}}}}}=-2.7673 \ [90 \% {{{{{\rm{HDI}}}}}}, -5.2846 \, {{{{{\rm{to}}}}}}-0.3959])$ but had no effect on reward/punishment learning rates or stimulus stickiness $(0\in 75 \% {{{{{\rm{HDI}}}}}})$ [33]. Simulation of the computational model retrodicted the comparable numbers of losses (Supplementary Result 1 and Supplementary Fig. 8) and the trial-by-trial average probability of choosing the optimal stimulus (Fig. 4C).

## Discussion

We have demonstrated some converging effects of a range of manipulations of 5-HT across both rats and humans which support its evolutionarily conserved role in behavioral flexibility and plasticity. Applicability of the winning models to these experiments was demonstrated by several validity checks including model fitness, model simulation and parameter recovery. Moreover, estimated model parameters had significant associations with conventional behavioral task measures (Supplementary Result 2 and Supplementary Tables 5–7), and the models performed similarly in terms of predicting the trial-by-trial choices in the serotonin manipulation conditions and their control conditions (Supplementary Table S8). Computational modelling indicated decreases or increases in choice repetition (‘stickiness’) or reinforcement learning rates following manipulations intended to decrease or increase serotonin function, respectively. Stickiness, a basic tendency to persevere versus ‘explore’, was modulated quite consistently in five serotonergic manipulations examined across both rats and humans. Stickiness was decreased by neurotoxic 5-HT depletion in rats and by acute 1 mg/kg SSRI in rats (citalopram) and healthy humans (20 mg escitalopram), treatments presumably reducing 5-HT signalling, based on evidence cited in the Introduction. By contrast, stickiness was increased (at 75% HDI) following both repeated (5 mg/kg for 7 days) and further sub-chronic (10 mg/kg twice a day for 5 days) dosing of SSRI in rats, treatments probably boosting 5-HT function, although stickiness was not increased by chronic escitalopram in humans.

The findings on stickiness were apparently different from those produced by tryptophan depletion in humans, which correlated with persistent activity in fronto-striatal regions [55]. 5-HT depletion from orbitofrontal cortex in the marmoset may have similar effects, opposite from those observed following 5-HT depletion of the amygdala [46]. Thus, 5-HT modulation of different brain regions can have opposing influences on such parameters.

Learning rates were also modulated by five serotonergic manipulations across species, although these effects were less consistent. Reward learning rate increased after sub-chronic citalopram (5 mg/kg for 7 days followed by 10 mg/kg twice a day for 5 days) in rats compared with the vehicle group, presumably boosting 5-HT neurotransmission. Conversely, humans given a single 20 mg dose of escitalopram, possibly reducing post-synaptic serotonin signaling [56], and rats receiving 5,7-DHT to reduce forebrain 5-HT, had decreased reward learning rates, consistent with some other findings and theories in the literature that 5-HT plays an important role in reward learning [55, 57–59]. This parallels the reduction of reinforcement learning rates following 5,7-DHT infused directly in the marmoset amygdala or OFC to produce local 5-HT depletion [46]. However, less consistent with the findings above were effects of acute low and high dose citalopram in rats. Although their opposite effects were consonant with presumed differing actions on 5-HT function, they were inconsistent with those obtained following repeated and chronic administration. Moreover, repeated and chronic citalopram in rats both increased punishment learning rate markedly, which was not seen with any other 5-HT manipulation.

Collectively, the present and the previous results show that serotonin has some common actions on latent computational mechanisms, especially those supporting flexible decision-making and plasticity in rats, marmoset monkeys and humans.

Stickiness, the only value-free parameter in our reinforcement learning model, contributed to a core feature of complex behavior, i.e. exploration. Lower stickiness, even negative stickiness, is generally associated with more exploratory behavior, which however is not a unitary construct [60]. Exploratory behavior can reflect directed information gathering, but at another level it can be mechanistic or rigid, resulting from ‘decisional noise’, producing apparently flexible behavior but, in fact, representing a fundamental performance heuristic recruited in volatile settings [61]. Another potential measure of exploration is reflected in reinforcement sensitivity, which can be interpreted as reflecting the balance between exploiting and exploring tendencies (low reinforcement sensitivity is sometimes referred to as ‘random exploration’) [61].

Whilst the effects of serotonin on reinforcement sensitivity revealed by the present analyses were ostensibly more difficult to interpret—underscoring that stickiness is a distinct mechanism—there is an intriguing parallel with a recent study. Langley et al. [33] have recently shown diminished reinforcement sensitivity in healthy humans following chronic escitalopram (20 mg) performing the same PRL task and modelled identically—this reduction hence being in the same direction as for the acute dosing in humans and sub-chronic dosing in rats. Although this similarity in effects of single and chronic dosing in humans was paradoxical and unexpected, reinforcement sensitivity in rats following sub-chronic dosing was also decreased. These effects of reduced reinforcement sensitivity (value-based) may correspond to what has been termed “emotional blunting” or “SSRI-induced apathy syndrome” in patients with MDD [33, 62–64]. A reduction in inverse temperature can also be interpreted as a reduction in “maximisation” of reinforcement and this is a shift in the balance between “exploitation” and “exploration” [60]. However, it is evident that this drift to exploration is not always accompanied by reduced “stickiness”, indicating different processes underlying choice variability.

The present analyses focusing on behavioral flexibility are relevant to current hypotheses of actions of psychedelic agents such as psilocybin and LSD on neuronal plasticity and cognitive flexibility [65, 66]. Whilst LSD is mostly known for its 5-HT_2A_ agonist properties, it is also a 5-HT_1A_ agonist and suppresses dorsal raphe serotonin neuron activity [67]. Indeed, lysergic acid diethylamide (LSD) was recently shown to reduce stickiness during PRL performance of healthy humans [17], which aligns with 5-HT_1A_ somatodendritic autoreceptor effects associated with the reduced stickiness shown here following acute SSRI in humans and low dose SSRI in rats. LSD also markedly increased reinforcement learning rates for both reward and punishment [17], which were also increased following sub-chronic SSRI dosing in rats. This may agree with reports that optogenetic stimulation of 5-HT neurons in the dorsal raphe increased reinforcement learning rates [68]. Indeed, the 5-HT_2A_ receptor is involved in plasticity [69, 70] and associative learning [71]. Furthermore, during initial learning (pre-reversal), LSD decreased reinforcement sensitivity [17], in line with the acute and chronic [33] SSRI effects in humans and sub-chronic effect in rats.

Manifestation of high or low stickiness may bear on the neural representation of discrete states of the world. In the context of PRL, for example, one state would be “option A is mostly correct” (pre-reversal) whilst another state would be “option B is mostly correct” (post-reversal). To perform well during PRL, in this view, veridical state representations inferred by the brain are critical, as are veridical probabilities of transitions between states. Indeed, the OFC is implicated in representing states [72, 73]. One possibility, therefore, is that these results concerning stickiness collectively reflect an influence of serotonin on inferring states or state transitions. This would align with recent theorising on OCD (where stickiness is low during PRL) [7], which posits that the disorder can be characterised by excessive statistical uncertainty (variance, or inverse precision) about the probability of transitions between states (e.g. from the state of dirty hands to clean hands after washing), particularly those that are action-dependent [74]. The optimal response to uncertainty about the current state would be exploratory behavior to continue gathering information [74]. SUD (where stickiness is high) [7], meanwhile, may be characterised by over-encoding of state-specific rules and information [75]. The model of state transition uncertainty can explain excessive behavioral switching (i.e. low stickiness) as well as heightened perseveration (i.e. high stickiness) and can be extended to other conditions including generalised anxiety disorder, autism spectrum disorder (ASD), and schizophrenia [74].

Dose-dependent effects of SSRIs are key to understanding serotonin function in this cross-species analysis. Acute low- and high-dose SSRI administration lowered and increased stickiness, respectively, which likely reflected sensitive measures of opposite effects on 5-HT activity. Evidence from positron emission tomography (PET) imaging has shown that acute SSRI in humans, at the dose used here, lowers 5-HT concentrations in projection regions [56], although there can be considerable individual differences in this action—which may relate to the variability in the reinforcement sensitivity parameter evident in Fig. 4. The reduction in 5-HT levels in terminal projection areas is believed to reflect the activation of 5-HT_1A_ autoreceptors by increases in extracellular serotonin following reuptake inhibition, which in turn leads to decreased firing rates of 5-HT neurons [27, 76]. We posit that the high acute dose of SSRI used in rats, which heightened stickiness, overcame 5-HT_1A_ autoreceptor-mediated regulation [29] in terminal projections, although this action shows considerable regional variation [77]. We did not actually measure 5-HT levels after acute low and high doses of the SSRI and so this conjecture remains speculative.

The dose-dependent effects on stickiness may have implications for the treatment of OCD in particular, one of numerous conditions for which SSRIs are first-line pharmacotherapy [25, 26, 78, 79]. One puzzle has been why doses up to three times higher than those used in MDD are optimal for reducing symptoms of OCD [80]. In fact, guidelines for OCD recommend titrating to the maximum approved dose [81], yet using these high doses in MDD does not improve efficacy and instead increases side-effects [80]. That both the repeated 5 mg/kg SSRI and the sub-chronic 10 mg/kg treatments in rats increased stickiness in the present study may be relevant for understanding this clinical phenomenon.

Notably, the computational models demonstrated better prediction in humans versus rats; indeed, prediction in humans was in many cases excellent with little margin for improvement (Supplementary Table S8). The lesser (but still good) performance in rodents might in principle reflect an additional uncaptured psychological process in rats (absent in human performance) but a more likely explanation is of higher levels of randomness in rat behaviour.

## Conclusion

It is imperative to relate animal and human experiments to improve models of psychiatric disorder and drug development [82–84]. Here, we provide evidence that serotonin can modulate fundamental components of learning important for plasticity (reinforcement learning rates) and especially behavioral flexibility (stickiness) in often similar fashion in rodents and humans. These findings indicate that serotonin’s modulatory influence on basic tendencies to persevere or explore is conserved across species and is thus of evolutionary significance. The effects of SSRIs on plasticity and flexibility are relevant for the pathophysiology and treatment of OCD and SUD, where parallel learning processes have been perturbed [7], and have implications for a wide range of other neuropsychiatric disorders, including depression [8, 9] and schizophrenia [38, 85].

### Supplementary information

Supplemental Materials
