# Supplementary material for: Comparable roles for serotonin in rats and humans for computations underlying flexible decision-making
Source: Neuropsychopharmacology. 2023 Nov 1;49(3):600–8. doi: 10.1038/s41386-023-01762-6 (PMC10789782; doi:10.1038/s41386-023-01762-6)
Supplement: Supplementary file 1 — Supplemental Materials [file 41386_2023_1762_MOESM1_ESM.docx]

**Comparable roles for serotonin in rats and humans for computations underlying flexible decision-making**

**Supplemental Materials**

Qiang Luo, *et al*.

Contents

[Supplementary Method 1: Probabilistic reversal learning task in rats and humans 2](#_Toc146173492)

[Supplementary Method 2: Experimental details of the 5-HT manipulations in rats and humans 4](#_Toc146173493)

[Supplementary Method 3: Model definitions 6](#_Toc146173494)

[Supplementary Result 1: Comparisons between model simulation and empirical data 10](#_Toc146173495)

[Supplementary Figure 1. Group comparison of conventionally behavioural measures averaged over 7 sessions for the depletion experiment in rats. 13](#_Toc146173496)

[Supplementary Figure 2. Changes of the probabilities of repeating a choice following the serotonin depletion in rats. 14](#_Toc146173497)

[Supplementary Figure 3. Comparison between empirical results and model simulations for the serotonin depletion experiment of rats. 15](#_Toc146173498)

[Supplementary Figure 4. Comparison between empirical results and model simulations for the acute high/low-dose SSRI experiments of rats. 16](#_Toc146173499)

[Supplementary Figure 5. Comparison between empirical results and model simulations for the repeated SSRI experiment of rats. 17](#_Toc146173500)

[Supplementary Figure 6. Comparison between empirical results and model simulations for the sub-chronic SSRI experiments of rats. 18](#_Toc146173501)

[Supplementary Figure 7. Comparison between empirical results and model simulations for the acute SSRI experiments of healthy humans. 19](#_Toc146173502)

[Supplementary Figure 8. Comparison between empirical results and model simulations for the chronic SSRI experiments of healthy humans. 20](#_Toc146173503)

[Supplementary Table 1. Prior distributions for model parameters 21](#_Toc146173504)

[Supplementary Table 2. Statistics for model comparisons. 22](#_Toc146173505)

[Supplementary Table 3. Mean estimations of the model parameters given by Model 2. 23](#_Toc146173506)

[Supplementary Table 4. Simulations for parameter recovery of the parameter values estimated for each group. 24](#_Toc146173507)

[Supplementary Table 5. Correlations between model parameters and conventional measures in rats: SSRI experiments. 25](#_Toc146173508)

[Supplementary Table 6. Correlations between model parameters and conventional measures in rats: repeated 5mg/kg citalopram experiment. 26](#_Toc146173509)

[Supplementary Table 7. Correlations between model parameters and conventional measures in humans: SSRI experiment. 27](#_Toc146173510)

[Supplementary Table 8. Model performances for two conditions in each experiment. 28](#_Toc146173511)

[Reference 29](#_Toc146173512)

**Supplementary Method 1: Probabilistic reversal learning task in rats and humans**

**Probabilistic reversal learning task: humans**

The task used in the human SSRI experiment [1] is shown in Figure 1A, and contained 80 trials: 40 during acquisition and 40 following reversal. In other words, there was a fixed number of trials and a single reversal. For the first 40 trials, one option yielded positive feedback on 80% of trials, the other option on 20% of trials. These contingencies reversed for the latter 40 trials. Positive feedback was given in the form of the word “CORRECT” on the touchscreen computer and a high tone, negative feedback was conveyed by the word “WRONG” and a low tone. The task was self-paced.

**Probabilistic reversal learning task: rats**

Following training and determination of stable levels of accuracy and a lack of side bias [2] in operant chambers controlled by the Whisker control system [3], rats were presented with two apertures illuminated simultaneously to the left and right of a central (inactive) aperture (Figure 1B). Responding at the ‘correct’ location was associated with an 80% probability food reward (and 20% probability of a time-out punishment), whereas responding at the ‘incorrect’ location yielded reward on only 20% of trials (and punishment on 80%). Reward was in the form of a 45 mg food pellet (Noyes dustless pellets; Sandown Scientific, Middlesex, UK) delivered to a food magazine positioned on the opposite wall of the operant chamber. Punishment was given in the form of a 2.5-second time-out. The left and right apertures were illuminated for 30 seconds signifying the response window. The next trial was triggered by retrieval of the pellet from the magazine. If no response was made, the trial was categorised as an omission and resulted in a 5-second time-out. Responding to an unlit aperture had no programmed consequence. Reversals occurred after the animal made eight consecutive correct responses, at which point the correct aperture became the incorrect aperture and vice versa. A session consisted of 200 trials to be completed during a 40-minute period. One session was conducted per day.

**Supplementary Method 2: Experimental details of the 5-HT manipulations in rats and humans**

**5,7-DHT forebrain 5-HT depletion: rats**

Sixteen rats were included in the final analysis. Rats were pre-treated intraperitoneally (i.p.) with 20 mg/kg of desipramine hydrochloride (Sigma, Poole, UK) in order to preserve noradrenergic neurons. Half of the rats were randomly assigned to receive bilateral intracerebroventricular (i.c.v.) infusions of 80 μg 5,7-DHT creatinine sulfate diluted in 10 μg of 10% ascorbic acid in saline, guided by a stereotaxic frame, whilst the other half received a sham infusion of 10 μg 0.01 M phosphate-buffered saline (PBS) – vehicle [2]. Post-mortem neurochemistry confirmed that 5,7-DHT infusions produced a near-total depletion of brain serotonin and decreased levels of the serotonin metabolite 5-hydroxyindoleacetic acid (5-HIAA) relative to controls in all regions examined: OFC, prelimbic cortex, anterior cingulate cortex, nucleus accumbens, dorsomedial striatum, dorsolateral striatum, amygdala, dorsal hippocampus (all p<.05)[2]. Levels of dopamine, norepinephrine, and the dopamine metabolite dihydroxyphenylacetic acid (DOPAC) were not significantly different from controls in any of these regions (all p > .05)[2]. Data were analysed from seven consecutive sessions conducted following surgery in the previous report [2]. Computational model convergence was achieved when modelling behavior from all seven sessions collectively, which is reported in the current study. Conversely, computational model convergence could not be achieved when modelling the seven sessions separately.

**SSRI administration: rats**

Animals were divided into groups matched for task accuracy and then randomly assigned via a Latin square design to receive injections i.p. of either citalopram or hydrobromide (1 mg/kg or 10 mg/kg; Tocris, Bristol, UK). Citalopram, dissolved in 0.01 M PBS, or vehicle was administered 30 minutes before the task [2]. Eleven rats were included in the final analysis after receiving vehicle, 1 mg/kg, or 10 mg/kg citalopram [2]. Fourteen rats were included in the repeated and sub-chronic citalopram experiment. The citalopram group was administered 5 mg/kg citalopram 30 min before testing, for seven consecutive days (*n*=7). The vehicle group (*n*=7), instead, received the same number of daily injections of 0.01 M phosphate-buffered saline [2]. After seven days, the citalopram group received 10 mg/kg of citalopram twice a day (about 4 h before the testing) for five consecutive days, to study the long-lasting effects of sub-chronic dosing [2].

**SSRI administration: humans**

The protocol was ethically approved (Cambridge Central NHS Research Ethics Committee, reference 15/EE/0004). Volunteers gave informed consent and were paid. Participants were healthy and without a personal or family history of psychiatric or neurological disorders [1]. In a randomised, double-blind, placebo-controlled, between-groups design [1], healthy volunteers received either escitalopram (*n*=32) or placebo (*n*=33). The PRL task was conducted following a 3-hour waiting period after oral drug administration to attain peak plasma escitalopram concentration [4]. Plasma analysis (*n*=59) verified increased escitalopram concentration [1] at 2.5 hours after the dose (t_54_ = 18.835, p < 0.001, mean = 14 ng/ml, standard deviation [SD] = 5.72) just before the task administration, and at 5.5 hours (t_54_ = 20.548, p < 0.001, mean = 17.24 ng/ml, SD = 4.27). Mood ratings were unaffected by single dose escitalopram administration (p > .05). There were no differences between groups in age, sex, years of education, depressive symptoms, or trait anxiety (all p > .05).

**Supplementary Method 3: Model definitions**

Model 1 incorporated three parameters and was used to test the hypothesis that 5-HT would affect how positive versus negative feedback guides behavior. Separate learning rates for positive feedback (reward) *α^rew^* and negative feedback (nonreward/punishment) *α^pun^* were implemented. Positive reinforcement led to an increase in the value *V_i_* of the stimulus *i* that was chosen, at a speed governed by the *reward learning rate* *α^rew^*, via $V_{i,t+1}\leftarrow V_{i,t}+ \alpha^{rew}(R_{t} - V_{i,t})$. *R_t_* represents the outcome on trial *t* (defined as 1 on trials where positive feedback occurred), and $(R_{t} - V_{i,t})$the prediction error. On trials where negative feedback occurred $R_{t}=0$, which led to a decrease in value of *V_i_* at a speed governed by the *punishment learning rate* *α^pun^*, according to $V_{i,t+1}\leftarrow V_{i,t}+ \alpha^{pun}\left( R_{t} - V_{i,t} \right).$ Stimulus value was incorporated into the final quantity controlling choice according to $Q_{t}^{reinf} = \tau^{reinf}V_{t}$. The additional parameter *τ^reinf^*, termed *reinforcement sensitivity*, governs the degree to which behavior is driven by reinforcement history. The quantities *Q* associated with the two available choices, for a given trial, were then input to a standard softmax choice function to compute the probability of each choice:

$P\left( \text{action}_{a} \right)=\text{softmax}_{\beta}^{a}\left( Q_{1}\text{...}Q_{n} \right)=\frac{e^{\text{βQ}_{a}}}{\sum_{\text{k=}1}^{n} e^{\text{βQ}_{k}}}$,

for *n*=2 choice options. The probability values for each trial emerging from the softmax function (*i.e.*, the probability of choosing stimulus 1) were fitted to the subject’s actual choices (*i.e.*, did the subject choose stimulus 1?). Softmax inverse temperature was set to *β*=1, and as a result the reinforcement sensitivity parameter (*τ^reinf^*) directly represented the weight given to the exponents in the softmax function.

Model 2 was as model 1 but for the human experiments incorporated a “stimulus stickiness” parameter *τ^stim^*, which measures the tendency to repeat a response to a specific perceptual stimulus, irrespective of the action’s outcome. For the rat experiments a “side (location) stickiness” parameter *τ^loc^* was substituted, which measures the tendency to repeat a response to a specific aperture in the operant chamber. Incorporating these two different stickiness parameters, depending on the species, accounts for task differences between the human and rat PRL experiments. This four-parameter model served to test whether accounting for stimulus-response learning, in addition to learning about action-outcome associations, would best characterise behavior. The stimulus stickiness effect was modelled as $Q_{t}^{stim}=\tau^{stim}s_{t-1}$, where $s_{t-1}$ was 1 for a stimulus that was chosen on the previous trial and was otherwise 0. The final quantity controlling choice incorporated this additional parameter as ${{Q_{t}=Q}_{t}^{reinf}+Q}_{t}^{stim}$. Quantities *Q*, corresponding to the two choice options on a given trial, were then fed into the softmax function as above.

Model 3 incorporated three parameters and served to test whether a single learning rate *α^reinf^*, rather than separate learning rates for rewards and punishments, optimally characterised behavior. Reward led to an increase in the value *V_i_* of the stimulus *i* that was chosen, at a speed controlled by the *reinforcement rate* *α^reinf^*, via $V_{i,t+1}\leftarrow V_{i,t}+ \alpha^{reinf}\left( R_{t} - V_{i,t} \right)$. *R_t_* represents the outcome on trial *t* (defined as 1 on trials where reward occurred), and $\left( R_{t} - V_{i,t} \right)$ the prediction error. On trials where punishment occurred *R_t_* = 0, which led to a decrease in value of *V_i_*. Model 3 also included the stimulus stickiness parameter. The final quantity controlling choice was determined by ${{Q_{t}=Q}_{t}^{reinf}+Q}_{t}^{stim}.$

Model 4 took a different approach, and had three parameters: *φ* (phi)*, ρ* (rho), and *β* (beta).

Derived from the experienced-weighted attraction model (EWA) of Camerer and Ho [53], here it was implemented as in den Ouden *et al*. [14], a study in which the EWA model best described behavior best on a nearly identical human task. A key difference to the other reinforcement learning models tested in this study is that here the learning rate can decline over time, governed by a decay factor *ρ* (rho). The EWA model weighs the value of new information against current expectations or beliefs, accumulated from previous experience.

Learning from reinforcement is modulated by an “experience weight”, *n_c,t_*, which is a measure of how often the subject has chosen a stimulus (*i.e.* experienced the action), and is updated every time the stimulus is chosen (where *c* is choice and *t* is trial) according to the experience decay factor *ρ* (range 0<*ρ*<1) and can increase without bounds [14]:

$$n_{c,t} \leftarrow n_{c,t-1}\rho+1.$$

The value of a choice is updated according to the outcome, *λ*, and the decay factor for previous payoffs, *φ* (range 0<*φ*<1) [14]

$$v_{c,t}\leftarrow(v_{c,t-1}\varphi n_{c,t-1}+\lambda_{t-1})/ n_{c,t}.$$

The payoff decay factor *φ* (phi) is related to a Rescorla–Wagner-style [54] learning rate *α* (as in Models 1-3), by $\alpha= 1 - \varphi$. A high value of *φ* means that stimuli keep a high fraction of their previous value and thus learning from reinforcement is slow. When *ρ* is high, then “well-known” actions (with high *n*) are updated relatively little by reinforcement, by virtue of the terms involving *n*, whilst reinforcement has a proportionately larger effect on novel actions (with low *n*). For comparison to Models 1-3, when $\rho=0$, the experience weight *n*, is 1, which reduces to a learning rate *α* controlling the influence of learning from prediction error. Choice in the EWA model is also governed by a softmax process, only here the softmax inverse temperature *β* was also a parameter able to vary, in contrast to Models 1-3.

In summary, the reward learning rate (*α^rew^*) indexed how quickly action value representation increased following a reward prediction error (when action outcome was better than predicted). Punishment learning rate (*α^pun^*) is an assay of the speed at which action value decreased following a punishment prediction error (outcome was worse than predicted). Stickiness measures a basic perseverative tendency: whether or not an action chosen on the previous trial was repeated, irrespective of its outcome. For rats, stickiness indexed the side (or location; *τ^loc^*) of responding whereas for humans, stickiness referred to (visual) stimulus stickiness (*τ^stim^*). Reinforcement sensitivity (*τ^reinf^*) measures the degree to which the values learned through reinforcement impact on choice behavior. Reinforcement sensitivity can be viewed as a value-based inverse temperature; stickiness as a value-free inverse temperature. Low values of stickiness or reinforcement sensitivity can be thought of as two different types of exploratory behavior; low reinforcement sensitivity represents exploration away from the more highly valued choice whereas low stickiness represents exploration away from the previously chosen stimulus or location irrespective of value.

**Supplementary Result 1: Comparisons between model simulation and empirical data**

*Simulation for serotonin depletion in rats*. We simulated the behaviour of 40 virtual rats in each group for 200 trials using the wining model (i.e. Model 2). We found that 5-HT depletion via 5,7-DHT significantly reduced the number of reversals completed (W = 1292.5, p = 5.6×10^-7^, by the Wilcoxon Rank Sum test for group difference in mean), reduced the win-stay rate (W=1347.5, p=1.4×10^-7^) and increased the lose-shift rate (W=415.5, p=2.2×10^-4^). These findings were consistent with the results from the conventional analysis reported in Bari et al. (2010).

*Simulation for acute SSRI in rats.* Again, we simulated 40 rats for each of the three groups for 200 trials using the winning model (i.e. Model 2). Compared with the low-dose (1 mg/kg) group, the high-dose (10 mg/kg) group completed more reversals (W=581.5, p=0.0333) and reduced the lose-shift rate (W=1284, p<10^-9^). These findings were consistent with the results from the conventional analysis reported in Bari *et al*. (2010).

*Simulation for repeated and sub-chronic SSRI in rats.* We simulated 40 rats for each of the 4 groups (repeated 5 mg/kg citalopram vs. vehicle, and sub-chronic 10 mg/kg citalopram vs. vehicle) for 200 trials using the winning model (i.e. Model 2). Compared with the vehicle group, the repeated 5 mg/kg citalopram group completed more reversals (W=412.5, p=0.0001) and had an enhanced win-stay rate (W=292, p=1.04×10^-6^). Using conventional analyses, Bari *et al*. (2010) had reported that the win-stay rate was significantly enhanced in the citalopram group compared with the vehicle group, while the increase in the number of reversals was only at a trend level. For the sub-chronic dosing group, the conventional analysis of the experimental data showed a significant increase in the number of reversals completed but no significant change in either the win-stay or the lose-shift rates when compared with the vehicle group (Bari *et al*. 2010). However, in the simulation, we found no significant change in these conventional measures (p>0.05).

*Simulation for acute SSRI in humans.* We simulated 40 virtual human participants for 80 trials (the reversal happened on the 41^st^ trial), and found a lower win-stay rate (W= 4916, p=4.32×10^-9^) and higher lose-shift rate (W=1492, p= 5.5×10^-9^) in the SSRI group compared with the placebo group. Consistent with the data reported originally by Skandali et al. (2018), in the simulation, the number of errors increased after SSRI administration (W=2556, p=0.0275) and this was particularly true during the acquisition phase of the task (W=2556, p=0.0271).

*Simulation for chronic SSRI in humans.* We simulated 40 virtual human participants for 80 trials (the reversal happened on the 41^st^ trial), and found a lower win-stay rate (W=4148, p=0.00004) and higher lose-shift rate (W=2258, p=0.0013) in the SSRI group compared with the placebo group. Consistent with the data reported originally by Langley et al (2022), in the simulation, no differences in the number of errors in the SSRI group compared with the control group.

**Supplemental Result 2: Relationship between model parameters and conventional behavioral measures**

Significant correlations between model parameters and the conventional measures were identified following false discovery rate (FDR). For the rat experiments, we considered the win-stay (proportion of trials where the subject stayed with the same choice following a reward), lose-shift (proportion of trials where the subject shifted choice following punishment), and number of reversals completed [20]. Win-stay and lose-shift were also examined in the human studies, as was perseveration [18].

In the human SSRI acute experiment, stimulus stickiness was positively correlated with win-stay rate (r=.51, p=.0066 on placebo; r=.62, p=.0005 following escitalopram) and also negatively correlated with lose-shift rate (r=-.63, p=.0003 on placebo; r=-.78, p=7.95×10^-7^ following escitalopram). In rats, side (location) stickiness was negatively correlated with lose-shift rate following an acute 1mg/kg dose of citalopram (r=-.89, p=.006), and positively correlated with win-stay rate in the vehicle group in the repeated administration experiment (r=.95, p=.0065). Side (location) stickiness was also positively correlated with the number of reversals achieved during the repeated administration (r=.89, p=.0205 following 5mg/kg citalopram per day and r=.97, p=.0049 with the same number of daily injections of vehicle). More correlations are reported in the Supplementary Tables 5-7.

**Supplementary Figure 1.** Group comparison of conventionally behavioural measures averaged over 7 sessions for the depletion experiment in rats.

Upper row shows the data before outlier removal (the average number of reversals over 7 sessions less than 1). Two outliers were removed from the rats with depletion. Lower row shows the data after outlier removal.

**Supplementary Figure 2.** Changes of the probabilities of repeating a choice following the serotonin depletion in rats.

‘rept.rate’ stands for the probability of repeating a choice regardless of rewarded or not, ‘rept.reward.rate’ is the probability of repeating a choice following a reward, and ‘rept.nonreward.rate’ is the probability of repeating a choice following a nonrewarded trial.


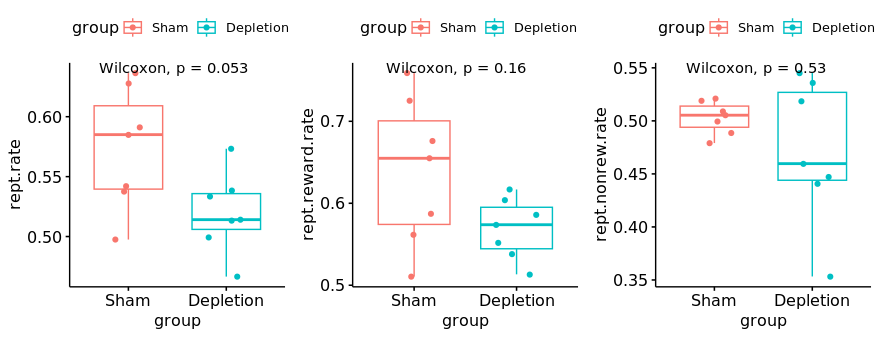


**Supplementary Figure 3.** Comparison between empirical results and model simulations for the serotonin depletion experiment of rats.


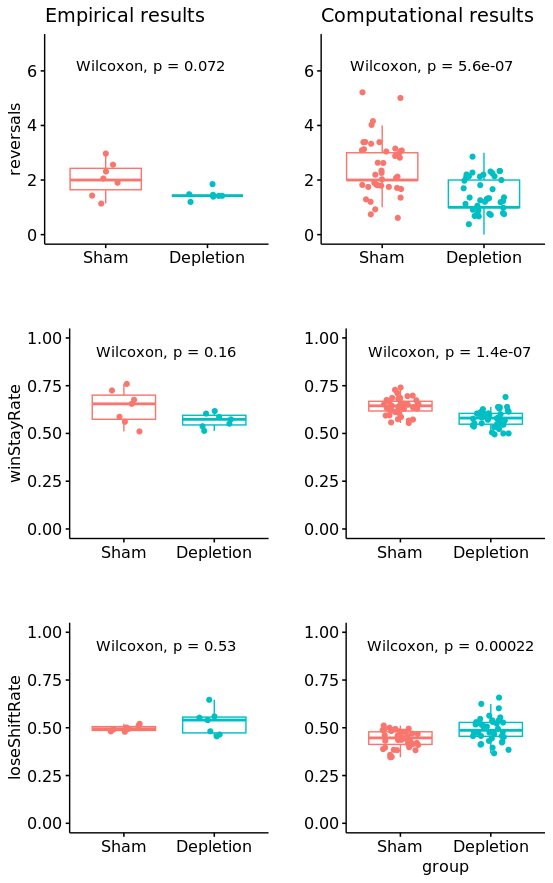


**Supplementary Figure 4.** Comparison between empirical results and model simulations for the acute high/low-dose SSRI experiments of rats.


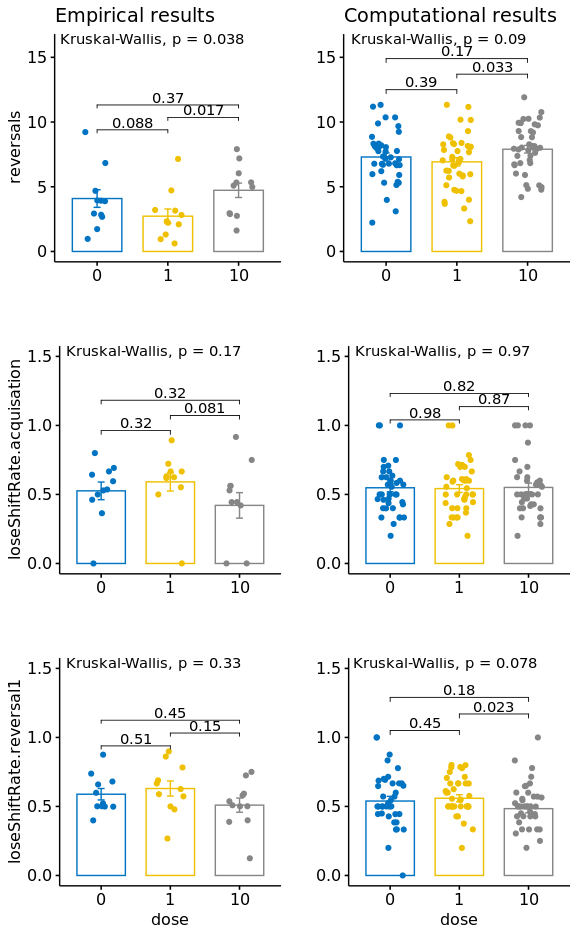


**Supplementary Figure 5.** Comparison between empirical results and model simulations for the repeated SSRI experiment of rats.


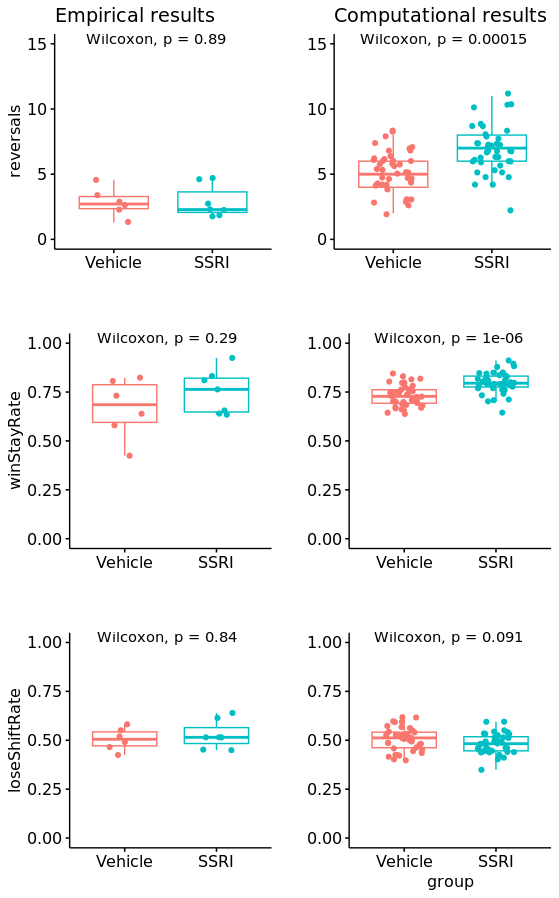


**Supplementary Figure 6.** Comparison between empirical results and model simulations for the sub-chronic SSRI experiments of rats.


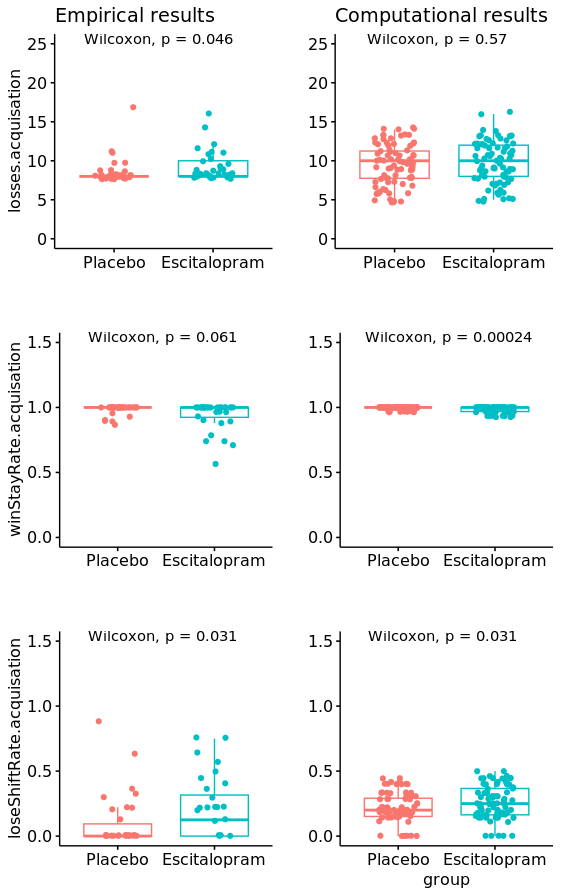


**Supplementary Figure 7.** Comparison between empirical results and model simulations for the acute SSRI experiments of healthy humans.

**
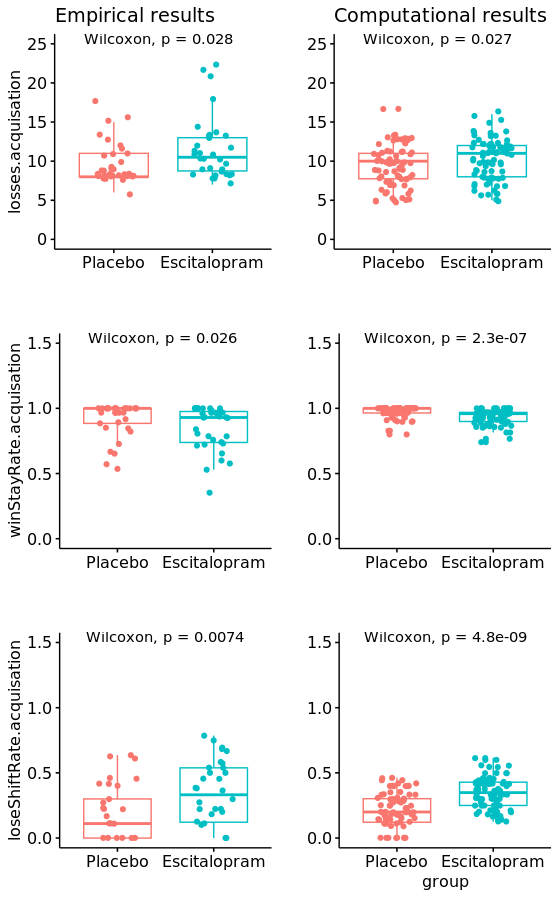
**

**Supplementary Figure 8.** Comparison between empirical results and model simulations for the chronic SSRI experiments of healthy humans.

**
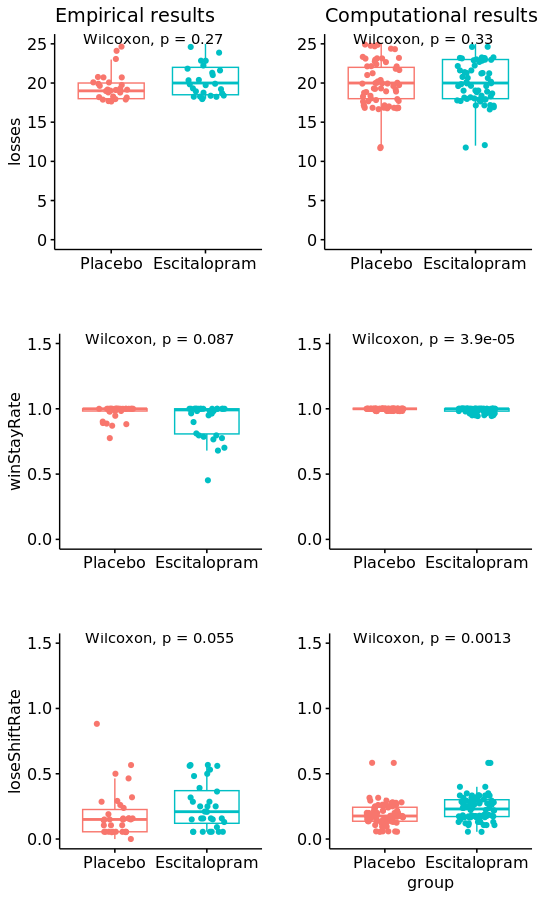
**

**Supplementary Table 1**. Prior distributions for model parameters

|  | **Models using each parameter** | **Prior** | **Reference** |
| --- | --- | --- | --- |
| **Model parameters** |  |  |  |
| reward learning rate, *α^rew^* | 1, 2 | Beta(1.2, 1.2) | den Ouden et al. (2013) |
| punishment learning rate, *α^pun^* | 1, 2 | Beta(1.2, 1.2) | den Ouden et al. (2013) |
| combined reward/punishment learning rate, *α^reinf^* | 3 | Beta(1.2, 1.2) | den Ouden et al. (2013) |
| reinforcement sensitivity, *τ^reinf^* | 1, 2, 3 | Gamma(*α*=4.82*, β*=0.88) | Gershman (2016) |
| stimulus stickness, *τ^stim^*  or side (location) stickness, *τ^loc^* | 2, 3 | Normal(0, 1) | Christakou et al. (2013) |
| experience decay factor, *ρ* | 4 | Beta(1.2, 1.2) | den Ouden et al. (2013) |
| decay factor for previous payoffs, *φ* | 4 | Beta(1.2, 1.2) | den Ouden et al. (2013) |
| softmax inverse temperature, *β* | 4 [note that *β =* 1 in all other models] | Gamma(*α*=4.82*, β*=0.88) | Gershman (2016) |
| **Intersubject variability in parameters** |  |  | – |
| Intersubject standard deviations for *α^rew^*, *α^pun^*, *α^reinf^, ρ , φ* | As above | Half-normal: Normal(0, 0.05) constrained to ≥0 | Kanen et al. (2019) |
| Intersubject standard deviations for *τ^reinf^, β* | As above | Half-normal: Normal(0, 1) constrained to ≥0 | Kanen et al. (2019) |

*rew* reward, *pun* punishment, *reinf* reinforcement, *stim* stimulus, *loc* location

**Supplementary Table 2.** Statistics for model comparisons.

| **Experiment** | **Rank** | **Name** | **max_rhat** | **log marginal likelihood** | **log posterior P(model)** |
| --- | --- | --- | --- | --- | --- |
| 5,7-DHT  (rats) | 2 | Model 1 | 1.5 | -14470.99 | -119.0791 |
|  | **1** | **Model 2** | 1.0 | -14351.91 | 0.0000 |
|  | 4 | Model 3 | 1.0 | -14592.29 | -240.3807 |
|  | 3 | Model 4 | 1.0 | -14508.16 | -156.2528 |
| Acute SSRI  (rats) | 4 | Model 1 | 1.0 | -3711.99 | -59.1169 |
|  | **1** | **Model 2** | 1.0 | -3652.87 | -0.0006 |
|  | 2 | Model 3 | 1.0 | -3660.24 | -7.3654 |
|  | 3 | Model 4 | 1.0 | -3709.78 | -56.9105 |
| Chronic SSRI  (rats) | 2 | Model 1 | 1.0 | -12391.85 | -235.9039 |
|  | **1** | **Model 2** | 1.0 | -12155.95 | 0.0000 |
|  | 4 | Model 3 | 1.0 | -12204.65 | -48.6963 |
|  | 3 | Model 4 | 1.0 | -12398.79 | -242.8457 |
| Sub-chronic SSRI  (rats) | 3 | Model 1 | 1.0 | -8735.83 | -59.8212 |
|  | **1** | **Model 2** | 1.0 | -8676.01 | -0.0005 |
|  | 2 | Model 3 | 1.0 | -8683.63 | -7.6163 |
|  | 4 | Model 4 | 1.7 | -8760.39 | -84.3796 |
| Acute SSRI  (humans) | 2 | Model 1 | 1.0 | -1771.95 | -7.6840 |
|  | **1** | **Model 2** | 1.0 | -1764.26 | -0.0005 |
|  | 4 | Model 3 | 1.0 | -1821.33 | -57.0660 |
|  | 3 | Model 4 | 1.0 | -1783.84 | -19.5782 |

﻿The model ranked 1st was the winning model. Model names and parameters correspond to Table 2. Log marginal likelihood and log posterior P (model) are comparison metrics used to determine the best model. A numerically larger (less negative) log marginal likelihood is better. *rew* reward, *pun* punishment, *reinf* reinforcement, *loc* location

**Supplementary Table 3.** Mean estimations of the model parameters given by Model 2.

| **Groups** | Reward learning rate, *α^rew^* | | Punishment learning rate, *α^pun^* | | Reinforcement sensitivity, *τ^reinf^* | | Stimulus stickness, *τ^stim^*  or side (location) stickness, *τ^loc^* | |
| --- | --- | --- | --- | --- | --- | --- | --- | --- |
|  | Mean  (sd) | 95% HDI | Mean  (sd) | 95% HDI | Mean  (sd) | 95% HDI | Mean  (sd) | 95% HDI |
| Sham-rats | 0.09  (0.02) | [0.04, 0.13] | 0.02  (0.01) | [1.03×10^-4^, 0.04] | 3.07  (0.41) | [2.22, 3.85] | 0.18  (0.07) | [0.05, 0.31] |
| 5,7-DHT-rats | 0.05  (0.02) | [0.01, 0.08] | 4.70×10^-3^  (0.01) | [3.48×10^-6^, 0.01] | 3.13  (0.38) | [2.39, 3.89] | -0.12  (0.06) | [-0.24, -0.01] |
| Vehicle-rats | 0.67  (0.08) | [0.51, 0.83] | 0.95  (0.04) | [0.88, 1.00] | 2.23  (0.39) | [1.48, 2.99] | 0.22  (0.08) | [0.06, 0.37] |
| 1mg/kg-rats | 0.88  (0.07) | [0.76, 1.00] | 0.95  (0.04) | [0.88, 1.00] | 2.15  (0.38) | [1.46, 2.93] | 0.03  (0.08) | [-0.12, 0.20] |
| 10mg/kg-rats | 0.52  (0.08) | [0.38, 0.67] | 0.91  (0.05) | [0.81, 1.00] | 2.52  (0.41) | [1.73, 3.30] | 0.29  (0.08) | [0.12, 0.45] |
| Placebo-human | 0.59  (0.07) | [0.46, 0.72] | 0.20  (0.03) | [0.15, 0.25] | 7.55  (0.88) | [5.91, 9.35] | 0.36  (0.09) | [0.18, 0.53] |
| SSRI-human | 0.39  (0.05) | [0.29, 0.49] | 0.21  (0.03) | [0.15, 0.27] | 5.86  (0.82) | [4.21, 7.46] | 0.18  (0.08) | [0.02, 0.34] |
| Vehicle-rats | 0.26  (0.07) | [0.11, 0.40] | 0.27  (0.11) | [0.06, 0.48] | 2.30  (0.41) | [1.50, 3.11] | 0.11  (0.09) | [-0.08, 0.28] |
| Repeated-rats | 0.35  (0.07) | [0.21, 0.51] | 0.60  (0.11) | [0.38, 0.81] | 2.21  (0.41) | [1.40, 2.99] | 0.27  (0.09) | [0.10, 0.44] |
| Vehicle-rats | 0.25  (0.06) | [0.13, 0.38] | 0.38  (0.10) | [0.19, 0.57] | 2.36  (0.30) | [1.78, 2.97] | -0.03  (0.07) | [-0.17, 0.11] |
| Sub-chronic-rats | 0.73  (0.08) | [0.56, 0.88] | 0.86  (0.09) | [0.69, 1.00] | 1.36  (0.23) | [0.92, 1.83] | 0.14  (0.07) | [0.01, 0.28] |

**Supplementary Table 4.** Simulations for parameter recovery of the parameter values estimated for each group.

| Group | Reward learning rate, *α^rew^* | Punishment learning rate, *α^pun^* | Reinforcement sensitivity, *τ^reinf^* | Stimulus stickness, *τ^stim^*  or side (location) stickness, *τ^loc^* |
| --- | --- | --- | --- | --- |
| 5,7-DHT | 97% | 97% | 100% | 97% |
| Sham | 97% | 97% | 90% | 97% |
| SSRI 1mg/kg | 97% | 97% | 97% | 97% |
| 10 mg/kg SSRI | 90% | 97% | 93% | 90% |
| Vehicle group | 97% | 90% | 97% | 93% |
| Chronic 5 mg/kg | 97% | 93% | 100% | 100% |
| Chronic sham | 97% | 97% | 93% | 83% |
| Sub-chronic 10 mg/kg | 97% | 93% | 93% | 93% |
| sub-chronic sham | 97% | 97% | 97% | 93% |
| SSRI in humans | 90% | 90% | 93% | 97% |
| Placebo in humans | 97% | 97% | 97% | 100% |

The estimated parameters were used to simulate the winning model for 100 virtual subjects. The reversal occurred on the 41^st^, of 80 trials. One choice resulted in reward on 80% of trials and the other choice resulted in reward on 20% of trials. The parameters were then fitted from these simulated data. If the 95% HDI of an estimation included the corresponding true value, then we counted this parameter recovery as a success. The models were simulated 30 times for each group. The success rates of the parameter recovery were reported.

**Supplementary Table 5.** Correlations between model parameters and conventional measures in rats: SSRI experiments.

|  |  | **Side (Location) Stickiness, *τ^loc^*** | **Reward Learning Rate, *α^rew^*** | **Punishment Learning Rate, *α^pun^*** | **Reinforcement Sensitivity, *τ^reinf^*** |
| --- | --- | --- | --- | --- | --- |
| **Win-Stay** | **Vehicle** | -- | -- | r = .87  p = .007 | r = .94  p = .001 |
|  | **1 mg / kg Citalopram** | -- | -- | r = .94  p = .001 | r = .84  p = .009 |
|  | **10 mg / kg**  **Citalopram** | -- | -- | r = .87  p = .007 | -- |
| **Lose-Shift** | **Vehicle** | -- | -- | -- | -- |
|  | **1 mg / kg Citalopram** | r = -.89  p = .006 | -- | -- | r = .85  p = .009 |
|  | **10 mg / kg Citalopram** | -- | -- | -- | -- |
| **Reversals** | **Vehicle** | -- | -- | -- | -- |
|  | **1 mg / kg Citalopram** | -- | -- | -- | -- |
|  | **10 mg / kg Citalopram** | -- | -- | -- | -- |

Statistics reported for correlations significant at p < .05 after correction for multiple comparisons. -- = not significant. *rew* reward, *pun* punishment, *reinf* reinforcement, *loc* location

**Supplementary Table 6.** Correlations between model parameters and conventional measures in rats: repeated 5mg/kg citalopram experiment.

|  |  | **Side (location) Stickiness, *τ^stim^*** | **Reward Learning Rate, *α^rew^*** | **Punishment Learning Rate, *α^pun^*** | **Reinforcement Sensitivity, *τ^reinf^*** |
| --- | --- | --- | --- | --- | --- |
| **Win-Stay** | **Vehicle** | r = .95  p = .0065 | r=.94  p=.0065 | -- | r=-.96  p = .0059 |
|  | **Repeated** | -- | r=.96  p=.0059 | r=.89  p=.0205 | -- |
| **Lose-Shift** | **Vehicle** | -- | -- | -- | -- |
|  | **Repeated** | -- | -- | -- | -- |
| **Reversals** | **Vehicle** | r = .97  p = .0049 | r = .09  p = .0181 | -- | r = -.92  p = .0150 |
|  | **Repeated** | r=.89  p=.0205 | -- | -- | -- |

Statistics reported for correlations significant at p < .05 after correction for multiple comparisons. -- = not significant. *rew* reward, *pun* punishment, *reinf* reinforcement, *loc* location

**Supplementary Table 7.** Correlations between model parameters and conventional measures in humans: SSRI experiment.

|  |  | **Stimulus Stickiness, *τ^stim^*** | **Reward Learning Rate, *α^rew^*** | **Punishment Learning Rate, *α^pun^*** | **Reinforcement Sensitivity, *τ^reinf^*** |
| --- | --- | --- | --- | --- | --- |
| **Win-Stay** | **Placebo** | r = .51;  p = .0066 | -- | r = .44;  p = .0217 | r = .90;  p = 6.05 × 10^-12^ |
|  | **Escitalopram** | r = .62;  p = .0005 | -- | -- | r = .93;  p = 2.99 × 10^-13^ |
| **Lose-Shift** | **Placebo** | r = -.63;  p = .0003 | -- | -- | r = -.91;  p = 5.53 × 10^-12^ |
|  | **Escitalopram** | r = -.78;  p = 7.95 × 10^-7^ | -- | -- | r = -.85;  p = 3.51 × 10^-9^ |
| **Perseveration** | **Placebo** | -- | -- | -- | r = .50;  p = .0078 |
|  | **Escitalopram** | -- | -- | -- | r = .45;  p = .0217 |

Statistics reported for correlations significant at p < .05 after correction for multiple comparisons. -- = not significant. *reinf* reinforcement, *stim* stimulus

**Supplementary Table 8.** Model performances for two conditions in each experiment.

| **Experiments** | **AUROC**  Sham / Treatment |
| --- | --- |
| Humans: chronic citalopram | 0.988 / 0.966 |
| Humans: 20 mg escitalopram | 0.955 / 0.917 |
| Rats: 1 mg/kg citalopram | 0.791 / 0.800 |
| Rats: 10 mg/kg citalopram | 0.791 / 0.793 |
| Rats: 5mg/kg citalopram  chronic | 0.692 / 0.734 |
| Rats: 10mg/kg citalopram  sub-chronic | 0.709 / 0.724 |
| Rats: neurotoxic depletion of 5-HT | 0.667 / 0.644 |

NOTE: To establish the degree to which models captured the empirical data in different conditions, we calculated the area under the receiver operating characteristic (AUROC) curve. Comparing the model predictions with the actual trial-by-trial choices, the AUROC values provide a well-understood metric for binary classification prediction (from 0.5 random prediction to 1.0 perfect prediction). The gap between model performance and perfection may represent either the potential for improved model performance, or true randomness that is intrinsically unpredictable.

**Reference**

1 Skandali N, Rowe JB, Voon V, Deakin JB, Cardinal RN, Cormack F, et al. Dissociable effects of acute SSRI (escitalopram) on executive, learning and emotional functions in healthy humans. Neuropsychopharmacology. 2018;43(13):2645-51.

2 Bari A, Theobald DE, Caprioli D, Mar AC, Aidoo-Micah A, Dalley JW, et al. Serotonin Modulates Sensitivity to Reward and Negative Feedback in a Probabilistic Reversal Learning Task in Rats. Neuropsychopharmacology. 2010;35(6):1290-301.

3 Cardinal RN, Aitken MRF. Whisker: A client-server high-performance multimedia research control system. Behavior Research Methods. 2010;42(4):1059-71.

4 Rao N. The clinical pharmacokinetics of escitalopram. Clinical Pharmacokinetics. 2007;46(4):281-90.
